# Not just a writer: PRC2 as a chromatin reader

**DOI:** 10.1042/BST20200728

**Published:** 2021-06-01

**Authors:** Michael Uckelmann, Chen Davidovich

**Affiliations:** 1 Department of Biochemistry and Molecular Biology, Biomedicine Discovery Institute, Faculty of Medicine, Nursing and Health Sciences, Monash University, Clayton, Victoria, Australia; 2 EMBL-Australia, Clayton, Victoria, Australia

**Keywords:** chromatin reader, DNA methylation, gene repression, histone modifications, polycomb repressive complex 2, PRC2

## Abstract

PRC2 deposits the H3K27me3 repressive mark, which facilitates transcription repression of developmental genes. The decision of whether a particular gene is silenced at a given point during development is heavily dependent on the chromatin context. More than just a simple epigenetic writer, PRC2 employs several distinct chromatin reading capabilities to sense the local chromatin environment and modulate the H3K27me3 writer activity in a context-dependent manner. Here we discuss the complex interplay of PRC2 with the hallmarks of active and repressive chromatin, how it affects H3K27me3 deposition and how it guides transcriptional activity.

## Introduction

Chromatin structure and epigenetic context are crucial regulatory factors for gene expression in eukaryotes. Chromatin modifiers can shape chromatin structure and change epigenetic states in order to change gene expression profiles. These protein complexes have modular functional centers that provide chromatin ‘reader', ‘writer', and ‘eraser' functions [1]. Chromatin modifiers can sense a certain chromatin state through direct interaction with histone, DNA or RNA modifications or preference for DNA sequence features (*reading*). They can catalyze histone or DNA modifications (*writing*) and the removal of already present modifications (*erasing*) [1]. Repressive chromatin modifiers inhibit gene expression while activating modifiers promote gene expression. This review is focused on the chromatin *reading* functions of the repressive chromatin modifier polycomb repressive complex 2 (PRC2).

PRC2 is critical for the stable transcriptional repression of polycomb-target genes [2–6]. PRC2 is a modular multi-protein complex that combines chromatin reading and writing activities. The core PRC2 — including the subunits EZH1/2, SUZ12, EED and RBBP4/7 — can associate with different facultative subunits to modulate its functionality (reviewed in [4–6]). Some of the facultative subunits confer different chromatin reader functionality (more below).

As a chromatin writer, PRC2 catalyzes the mono-, di- and tri-methylation of lysine residue 27 on histone H3, giving rise to the H3K27me1, H3K27me2, and H3K27me3 epigenetic marks, with the latter facilitating transcriptional silencing [2–6]. While H3K27me3 is the prototypical PRC2 mark, H3K27me1/2 also have a distinct distribution but their function is poorly understood [7]. The different reader functionalities, conferred by PRC2 compositional diversity, allow PRC2 to sense genes that are marked for stable transcriptional silencing and to regulate its writing activity accordingly. Herein we review the most recent literature on this subject, which collectively suggests the following model: Hallmarks of active chromatin restrict the capacity of PRC2 to engage constructively with transcriptionally active loci, preventing H3K27me3 accumulation there. Certain repressive marks can stimulate PRC2 chromatin binding and catalysis, and thus trigger positive feedback loops to promote and maintain the repressed state of target genes.

## Positive feedback loops propagate H3K27me3 domain formation

PRC2 can recognize its own catalytic product, H3K27me3. Binding to H3K27me3 allosterically activates the methyltransferase activity of PRC2 [8–10]. This mechanism triggers a positive feedback loop that promotes the amplification and maintenance of the H3K27me3 mark at target sites [8–10]. The structural basis for this allosteric activation has been established [8,9,11]: The H3K27me3-modified lysine binds to an aromatic cage in the PRC2 subunit EED, which in turn leads to the stabilization of the catalytic center in its active conformation (reviewed in [5,12]). Notably, PRC2 can methylate specific lysines on JARID2 [13] and PALI1 [14], which can then bind to the same aromatic cage to trigger an allosteric activation [13,14].

The function of allosteric activation in H3K27me3 domain formation has been studied using mutations in the EED aromatic cage [15]. The authors propose a ‘hit-and-run' mechanism, based on the presence of H3K27me3 nucleation sites. After the removal of H3K27me3 in model cell lines, Oksuz et al. found that PRC2 initially catalyses H3K27me3 only at these nucleation sites. The facultative PRC2 subunits JARID2 and MTF2 facilitate the nucleation activity. Allosteric activation of PRC2 by H3K27me3 then initiates a positive feedback loop that allows the mark to spread into adjacent chromatin regions [15]. Such a spreading model is substantiated by *in vitro* data showing that PRC2 preferentially modifies neighboring nucleosomes, which requires its allosteric activation [16]. Cryo-EM studies provided a structural rationale for the propagation of H3K27me3 to neighbouring nucleosomes [17,18]. The structures show that PRC2 binds between two nucleosomes, where one of the nucleosomes provides the substrate lysine and the other provides the H3K27me3 allosteric effector.

## PRC1-driven H2AK119ub enhances H3K27me3 domain formation

Besides interacting with its own product, PRC2 also interacts with the catalytic product of the polycomb repressive complex 1 (PRC1) [19–24]. PRC1 ubiquitinates H2A at lysine 119 (H2AK119ub). This modification is essential for transcriptional repression of polycomb-target genes [19–21]. The H2AK119ub mark provides an additional positive feedback loop that helps to establish H3K27me3 at target genes during development [19–23]. This feedback loop is initiated through binding of H2AK119ub by the facultative PRC2 subunits JARID2 and AEBP2. Direct interaction of JARID2 with ubiquitinated H2A has been proposed based on cell biological and biochemical experiments [22,23]. This was confirmed by a recent cryo-EM structure, showing that both JARID2 and AEBP2 interact directly with the ubiquitin ligated to K119 of H2A [24]. Biochemically, the consequence of these interactions is the enhanced methyltransferase activity of PRC2 [23,24].

After initial recruitment to target genes, the synergy between PRC1 and PRC2, promoted by positive feedback loops between H2AK119ub and H3K27me3, maintains transcriptional repression. The mechanisms by which PRC1 and PRC2 are initially recruited to their target genes are still not fully understood. The following sections discuss evidence for a potential function of active chromatin modifications and DNA methylation in restricting PRC2 to its target genes by preventing its recruitment at other genomic contexts.

## H3k36me2/3 inhibits the catalytic activity of PRC2

Methylation of lysine 36 on H3 is associated with actively transcribed chromatin, and di- and tri-methylation of H3K36 (H3K36me2/3) inhibits PRC2 activity [25,26]. In vitro, at low salt concentrations and using nucleosome arrays as the substrate, the inhibition of PRC2 by H3K36me2/3 is a *k*_cat_ rather than a *K*_M_ effect, meaning catalysis is affected, rather than substrate binding [27]. A H3 interaction site was identified on the PRC2 subunit EZH2 and a possible mechanism for the inhibition was proposed [27]: A hydrophobic channel could accommodate the H3 tail and lead to a negatively charged pocket formed by E579 [27]. This pocket would function as a ‘sensing pocket’ for the methylation status at H3K36. Mutagenesis of residues surrounding the sensing pocket alleviated PRC2 inhibition by H3K36me3 [27]. Interestingly, one of those mutations (K634E) also occurs in patients with Weaver syndrome [27,28].

A cryo-EM structure of the catalytic core of PRC2 in complex with a di-nucleosome, determined using a PRC2–AEBP2 complex, showed that the H3 tail does interact with EZH2, however the residues around H3K36 could not be resolved [17]. A more recent structure of the catalytic core of PRC2, determined using the PRC2–PHF1 complex, allowed tracing of the relevant parts of the H3 tail [18] and contested the role of the proposed E579 H3K36me binding pocket. The structural arrangement reported for the H3 tail positions H3K36 at a distance of 19 Å away from EZH2 E579 [18]. Mechanistically, the authors show that unmodified H3K36 is necessary for orienting the H3 tail towards the EZH2 active site, thus activating catalysis of H3K27-methylation [18]. In agreement with the kinetic studies [27], the cryo-EM structure [18] implies that H3K36 methylation inhibits PRC2 through a disruption of the H3 tail substrate placement without disrupting nucleosome binding. Methylation of K36 has been proposed to disrupt the geometry that guides H3K27 towards the active site [18]. This only happens in the context of the nucleosome and not on H3-tail peptides in isolation, highlighting the importance of the chromatin context for the functionality of epigenetic marks.

A recent study highlighted the relevance of H3K36 methylation for PRC2 regulation in cells [29]. The authors analyzed recovery kinetics of the H3K27me3 mark after EZH2 inhibition. They found that the inhibition of EZH2 leads to widespread accumulation of H3K36me2 throughout genic and intergenic regions genome-wide, in addition to the elimination of H3K27me3. When removing EZH2 inhibition, recovery of H3K27 methylation was much delayed on the subset of histones that carried a pre-existing H3K36 methylation [29]. This highlights that the inhibitory effect that H3K36-methylation exerts towards PRC2 also applies in a physiological setting. Notably, the study also suggests that H3K27me3 has an antagonistic effect towards H3K36 methylation.

## Polycomb-like proteins as H3K36me readers?

The polycomb-like proteins PHF1, MTF2 and PHF19 (also termed PCL1, PCL2 and PCL3, respectively) are mutually exclusive subunits of PRC2. Both PHF1 and PHF19 have been shown to specifically bind to H3K36me3 *in vitro* via an aromatic cage that confers methyl-lysine binding specificity [30–35]. The Tudor domain of MTF2 can also bind H3K36me3 peptides [35–37], but with ∼3-fold lower affinity compared with PHF1 [36] or PHF19 [36,37], and structural differences between their aromatic cages have been proposed as a reason for the difference in affinity [36]. Experiments in mouse embryonic stem cells showed that PHF19 facilitates the recruitment of PRC2 to a subset of target genes and promotes H3K27me3 [34,35]. PHF19 loss-of-function phenotypes, including reduced PRC2 binding to chromatin, reduced frequency of H3K27me3 marks and reduced promoter silencing, could be rescued by a wild type PHF19 but not by a PHF19 aromatic cage mutants, which confirms a role for the aromatic cage in PHF19 function [34,35]. Similar dependencies have been described for PHF1 [30]. Collectively, these results led to the proposal of a model where H3K36me3 tails facilitate PCL-mediated recruitment of PRC2 [30–37]. Based on this model, H3K36me3 binds to the aromatic cage of PCLs and facilitates the recruitment of PRC2 to active genes that are to be repressed during the cell-differentiation process [30–37].

Of note, broad co-localisation of PHF1 and PHF19 [30–37] with H3K36me3 on chromatin has not been found in any of these studies [30–37]. Moreover, independent work showed that the inhibition of transcription is sufficient for PRC2 recruitment [38], suggesting that transcriptional shutdown precedes PRC2 engagement with chromatin. Furthermore, PHF1 and PHF19 also bind to H3K27me3, albeit with lower affinity than to H3K36me3 [30,31,34]. Interestingly, both PHF1 and PHF19 bind with high affinity to the H3K27-methylated histone H3 variant H3t (H3tK27me3) [31,37], a variant highly expressed in testis but also present in other cell types. PHF19 does co-localize with H3tK27me3 in cells and during different stages of spermatogenesis *in vivo* [31,37], which contrasts with the lack of colocalization with H3K36me3 [31,34]. Structural studies have shown that H3K36me3 and H3tK27me3 peptides are bound by the PHF1/19 Tudor domain in different orientations [37]. Curiously, the peptide backbones traverse the Tudor domain in opposite directions, meaning that if the Tudor domains are superimposed then the N-terminus of the H3tK27me3 peptide aligns with the C-terminus of the H3K36me3 peptide [37]. It remains to be determined how these opposite orientations of the H3 and H3t tails would affect binding in the context of chromatin.

The PRC2–PHF1 [39] and the PRC2–PHF19 [40] complexes bind DNA with low nanomolar affinity in the absence of the H3K36me3 mark and nucleosomes [41]. Hence, it would be important to determine to what extent the PCL–H3K36me3 interactions add to this in terms of affinity, specificity, and potentially the correct orientation of PRC2 on chromatin. Detailed structure-function works using PRC2–PCL bound to H3K36me3 and H3tK27me3 methylated chromatin are required to determine how, and to what extent, the PCL–H3K36me3 interactions facilitate the recognition of chromatin by PRC2.

## MLL2 competes with PRC2 to catalyze H3K4me2/3

MLL methyltransferases are parts of COMPASS complexes and catalyze the mono-, di- and tri-methylation of lysine 4 on H3 (H3K4me1/2/3), a histone modification associated with active genes (reviewed in [42]). While H3K4me3 inhibits PRC2 activity *in vitro* [24,25], it has been found to co-occur with H3K27me3 at specific regions of the genome. This gave rise to the concept of bivalent chromatin, associated with developmental genes, that can carry both active and repressive histone modifications (reviewed in [43]).

Recent findings clarified the interplay between the H3K4me3 methyltransferase MLL2 (KMT2B) and PRC2 in the regulation of bivalent developmental genes. MLL2 and PRC2 appear to interact competitively. Knockdown of MLL2 in embryonic stem cells leads to accumulation of polycomb group proteins and H3K27me3 at bivalent promoters [44]. As a consequence, these chromatin loci become less accessible, transcription is repressed and they migrate into the inactive compartment of the genome [44]. A recent study on genetic interactions in an MLL2-deficient background confirmed that knockout of MLL2 leads to accumulation of H3K27me3 and transcriptional repression at MLL2 target genes [45]. Simultaneous knockdown of SUZ12 in the MLL2-deficient background restores normal gene expression levels, showing that the inhibitory effect relies on a functional PRC2 complex. Interestingly, disturbance of the DNA-methylation machinery also rescues the effects of MLL2 deficiency and mirrors the PRC2-dependent rescue to some extent [45]. This led the authors to propose a multi-tiered model for transcriptional regulation by PRC2, MLL2, and DNA methylation: When MLL2 is present, the MLL2 COMPASS complex occupies that promoter and prevents PRC2-driven transcriptional repression [45]. The precise mechanisms of COMPASS-PRC2 competition remain unclear, but accumulating evidence suggests that H3K4me3 is not an instructive factor in transcriptional activation [45,46]. This suggests a competition for chromatin access between PRC2 and MLL2 COMPASS as the driving factor of transcriptional regulation at these promoters. Direct evidence for this model is still required.

While negative regulation of PRC2 at H3K4me3-decorated promoters could be attributed to the COMPASS machinery [45], structural and biochemical works proposed a direct effect [24,25]. In vitro histone methyltransferase and binding assays demonstrated that H3K4me3 modification inhibits the catalytic activity of PRC2 without reducing its affinity to nucleosomes [25]. In agreement with that, structures of PRC2 in complex with a H3K4me3-modified nucleosome revealed two conformations for the H3 tail. Only in one conformation the tail reaches into the catalytic site [24]. These studies imply that while an unmodified H3 tail is correctly presented to the catalytic site, H3K4me3-modified tails spend at least some time externally to the active site and, therefore, reduce the catalytic efficiency [24,25]. This mechanism is similar to that of H3K36me2/3-mediated inhibition of PRC2, as discussed above [18,25,27]. In conclusion, the inhibition of PRC2 by the H3K4me3 mark [24,25] and the competition between PRC2 to MLL for chromatin binding [45] negatively regulate PRC2.

## DNA methylation restricts the spread of H3K27me3 domains

5-methyl-cytosine (5mC) is a DNA methylation catalyzed by DNA (cytosine-5)-methyltransferases (DNMTs). 5mC is largely considered a repressive epigenetic modification that inhibits transcription of target genes (reviewed in [47]). In embryonic cells, H3K27me3 and 5mC are anti-correlated at CpG islands and PRC2 is recruited preferentially to non-methylated CpG islands [48–51]. Evidence suggests that PRC2 is confined to unmethylated CpG islands through its inability to penetrate into heavily CpG-methylated regions of the genome [45,52,53]. Specifically, ablation of DNA methylation causes H3K27me3 to spread into previously inaccessible surrounding genomic regions and away from the promoter. This leads to a dilution of PRC2 concentration at promoters, reduces H3K27me3 methylation at promoters and may subsequently lead to transcriptional activation [45,52,53]. These data suggest that PRC2 has the ability to sense, whether directly or indirectly, the DNA methylation status of target sites. How PRC2 avoids methylated CpG islands is unclear at the molecular level and the literature is at times contradictory.

One structural study suggests that non-methylated DNA is specifically recognized by the winged-helix motif of the polycomb-like proteins MTF2 and PHF1 [54]. Somewhat surprisingly, the interactions between these PCL proteins to DNA [54] appear completely different than reported for other winged-helix proteins, which utilize different surfaces for DNA binding (reviewed in [55,56]). The authors propose a critical role for MTF2 and PHF1 in PRC2 recruitment to target-genes based on specific recognition of unmethylated CpG islands [54]. A parallel independent study proposed a different binding mode, based on biochemical data, mutagenesis, high-resolution structure determination (albeit without DNA) and structure modeling [39]. The data supports a binding mode that more closely resembles that of canonical winged-helix motifs with no evidence for sequence-specific DNA binding of PRC2-PHF1 [39]. Others have reported a preference of PRC2 for DNA containing only GC sequences, compared with only AT sequences [41]. The same work showed that the PRC2-AEBP2 complex binds CpG DNA that is completely methylated with higher affinity compared with non-methylated DNA [41]. This finding contrasts the mutual exclusive occurrence of H3K27me3 and 5mC in development [45,52], but fits with evidence that CpG methylation enhances H3K27me3-deposition in cancer [57]. While some of these findings could be seen as contradictory, it is possible that some variations between observations are attributed to the experimental system used.

## Nucleosome occupancy and histone variants regulate H3K27me3 domain formation

The preferred binding of PRC2 to linker DNA *in vitro* [41] agrees with its exclusion from regions of high nucleosome occupancy in cells [38]. Furthermore, CpG islands are the preferred target of PRC2 and these sites show reduced nucleosome occupancy, compared with the rest of the genome [60–62]. Polycomb Response Elements (PRE) in drosophila are also regions of nucleosome depletion [63]. This combined evidence suggests that PRC2 preferentially binds regions of low nucleosome occupancy. In contrast, genomic regions with high levels of DNA methylation have high nucleosome occupancy [59,64,65].

In actively transcribed genes, nucleosomes are partially disrupted by the passing RNA polymerase II (PolII) but are immediately reassembled after PolII passage [66]. During reassembly, histones can potentially be exchanged for newly synthesized histones, but H3K36me3 inhibits this histone exchange [67]. This leaves well-phased and H3K36me3-positive nucleosomes in the wake of PolII transcription; a poor substrate for PRC2. Additionally, the affinity of PRC2 to RNA [68–71] is higher than its affinity to DNA [39,41] or nucleosomes [41], which further inhibits association with chromatin. Of note, the histone variant H3.3 is enriched at actively transcribed genes [72]. H3.3 affects the activity of PRC2 [73] and of the H3K36-specific methylase SETD2 [74]. In vitro, nucleosomes containing H3.3 inhibit the methyltransferase activity of PRC2, while nucleosomes containing H3.1 are better substrates for PRC2 [73].

Phosphorylation of a serine residue unique to H3.3 (S31) promotes SET2 activity *in vitro* [74]. In cells, H3.3 S31 phosphorylation leads to an increase in H3K36me3 levels and a decrease in H3K27me3 [74,75]. This suggests that histone composition at actively transcribed genes prevents polycomb domain formation. The mechanism could involve direct interference with PRC2 activity, or maintenance of the ‘on-state' of the gene by promoting SET2 activity and easing PolII passage.

PRC2 is preferentially recruited to CpG islands with low nucleosome density [38]. This observation contrasts with the higher methylation activity of PRC2 towards densely packed nucleosome arrays *in vitro* [76]. There is also data suggesting that transcription start sites with high levels of H3K27me3 have a high nucleosome density [64]. This suggests that H3K27me3 nucleation is dependent on a fine balance between PRC2 affinities for target sites, substrate availability, in the form of nucleosome density, and allosteric activation of PRC2 by PALI1, JARID2 and H3K27me3.

## PRC2 senses chromatin during the nucleation and spread of H3K27me3 domains

**Figure 1.**
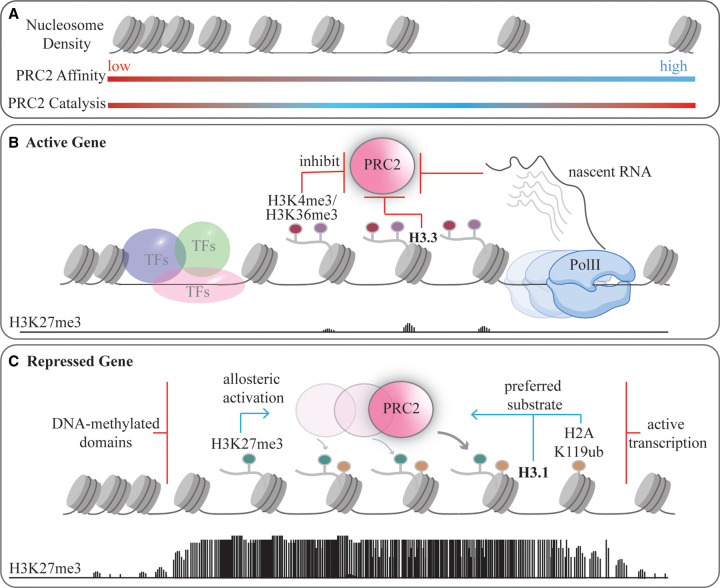
The chromatin context guides PRC2 activity towards its appropriate target sites. (**A**) Nucleosome occupancy can regulate PRC2 affinity for chromatin and PRC2 H3K27me3 methyltransferase activity. The binding preference of PRC2 for nucleosome-free linker DNA *in vitro* [41] and the anticorrelation of PRC2 binding and nucleosome density at CpG islands in cells [38] suggests highest affinity for regions of low nucleosome density. Increased nucleosome density, however, provides an increased density of the H3 substrate and, subsequently, the H3K27me3 effector. An intermediate nucleosome density may, therefore, allow for a good trade-off between substrate concentration and affinity and, subsequently, H3K27me3-induced allosteric activation [41,58]. (**B**) Hallmarks of active chromatin inhibit PRC2 activity. PRC2 is sequestered by RNA binding [41,68,79]. Free DNA at promoter regions is occupied by transcription factors and the histone methyltransferase activity of PRC2 is inhibited by the H3K36me3 [25,26] and H3K4me3 histone modifications [24,25], histone variant H3.3 [73] and RNA [78,80–83]. (**C**) Positive feedback loops auto-amplify H3K27me3 domain formation. H3K27me3 allosterically activates PRC2 [8–10]. H2A/H3.1 nucleosomes [73] and H2AK119-ubiquitinated nucleosomes [19–24] are preferred substrates for PRC2.

Several molecular ‘fail-safe' mechanisms prevent PRC2 from establishing H3K27me3 domains at actively transcribed genes. At genes marked for silencing, several positive feedback loops promote PRC2 activity (Figure 1).

At actively transcribed genes (Figure 1B), PRC2 can bind nascent RNA [68,77,78] that has been proposed to spatially remove PRC2 from chromatin [41,68,79] and to inhibit its methyltransferase activity [80–83]. The nucleosome-free region at the promoter is occupied by transcription factors, likely competing with PRC2 for DNA binding. Nucleosomes are disassembled in front of PolII but rapidly reincorporated after PolII [66] passage, limiting the pool of free DNA available for PRC2 binding at active genes. Sporadic interaction of PRC2 with nucleosomes is buffered by the presence of the H3.3 histone variant and H3K36me3, which both inhibit the histone methyltransferase activity of PRC2 [25,73], H3.3 phosphorylation further promotes active transcription and H3K36 methylation [74,75]. Collectively, all these PRC2-antagonising molecular cues are expected to prevent the nucleation of the H3K27me3 mark.

When transcription is shut off (Figure 1C), nascent RNA is no longer available to sequester PRC2. Transcription factors may dissociate from DNA, leaving stretches of naked DNA available for PRC2 binding. This would allow PRC2 to seed H3K27me3. This model agrees with the observation that pharmacological inhibition of PollI is sufficient to trigger the recruitment of PRC2 to target genes [38]. Importantly, recruitment alone is not sufficient to trigger an efficient H3K27me3 deposition because allosteric activation of PRC2 is necessary for that. Initial allosteric activation can be provided by the PRC2 subunits JARID2 [13] or PALI1 [14] in their methylated form. Once H3K27me3 is seeded it will keep PRC2 allosterically activated [9], regardless of the accessory subunits it harbors [80]. Optimal spacing between nucleosomes can further promote catalytic activity of PRC2 [41,58], possibly achieved with the aid of remodeling factors. At this point, the high local concentration of both the H3K27me3 effector and H3K27 substrate might counterbalance the less efficient recruitment of PRC2 seen at nucleosome-dense chromatin regions [38].

After being seeded, H3K27me3 can spread in three dimensions, allowing it to reach genomic regions that would otherwise be too distant [15]. The H3K27me3 spread would be limited by regions of very high nucleosome occupancy, as in the case of high 5mC [45,52,53,59,64] or constitutive heterochromatin [84]. The spread of the H3K27me3 mark is also limited by active chromatin marks like H3K36me3 [18,25–27,29] and H3K27ac [85], the transcriptional activity itself [38] and likely a gradual dilution of PRC2 concentration away from the initial seed. Further regulation of PRC2 recruitment is achieved through different PRC2 accessory subunits within the different subtypes of holo-PRC2 complexes.

## The chromatin reading activity of PRC2 is obstructed by oncohistones

The mechanisms discussed so far suggest that accurate transcriptional control relies on a tight balance between activating and repressive chromatin signals. Upsetting this balance can play an important role in cancer development and progression, which makes chromatin modifiers, including PRC2, attractive drug targets [86]. Here we discuss how oncohistones [87] affect the recruitment, enzymatic activity, and regulation of PRC2.

Histone variant H3.3 is mutated at residue K36 (K36M) in 95% of chondroblastomas [88]. This mutation inhibits H3K36me3 catalysis by SETD2 in a dominant fashion [89,90]. Structural studies suggest a mechanism where the H3K36M mutation leads to tight association of SETD2 with H3K36M mutant nucleosomes. As a consequence, SETD2 is sequestered on the mutant nucleosome, thus inhibiting H3K36 methylation *in trans* [91]. As discussed above, H3K36me2/3 inhibits the H3K27 methyltransferase activity of PRC2 [25–27] and restricts H3K27me3 spread externally from PRC2 target regions [29]. In agreement with this, the H3.3 K36M mutant facilitates spreading of the H3K27me3 mark into intergenic regions [90]. This reduces the levels of PRC1 at polycomb-target genes, leading to an unscheduled activation of gene expression [90].

Mutations of H3.3 at residue G34 are frequent in paediatric gliomas (G34V/R) [92,93] and giant cell tumor of the bone (G34W/L) [88]. This oncohistone mutation also impacts PRC2 recruitment indirectly, by interfering with H3K36 methylation. G34 mutations inhibit SETD2 activity, thereby reducing HK36me3 levels [94]. This causes recruitment of PRC2 to previously inaccessible enhancer elements. There, PRC2 catalyses H3K27me3 which silences the expression of genes regulated by these enhancers [94].

The H3K27M mutation is found in over 80% of diffuse intrinsic pontine gliomas (DIPG) cases [93]. H3K27M has a dominant negative effect on H3K27me3 levels: while only 2–17% of H3 molecules in DIPG cells carry the H3K27M mutation [95], global H3K27me3 levels are severely reduced [95–98]. The molecular mechanisms by which H3K27M exerts this dominant effect are still under debate. Initially, it was suggested that H3K27M inhibits PRC2 by sequestering it at non-target sites [95]. In agreement with this, PRC2 has higher affinity for H3K27M peptides compared with the wildtype sequence [96,99]. However, when incorporated into nucleosomes, the H3K27M mutation only increases PRC2 affinity modestly and even this modest increase is lost when measuring affinity for tri-nucleosomes [41]. This is in agreement with the finding that linker DNA is a major driver of PRC2 binding [41]. Furthermore, PRC2 is not generally colocalized with H3K27M *in vivo* [97,100] and H3K27M does not impair PRC2 recruitment [101]. Others suggest that PRC2 is selectively sequestered at specific enhancers [102]. Remodeling of the enhancer landscape has indeed been shown to be a feature of DIPG tumors [103].

Another work suggests that H3K27M has a long-lasting effect on PRC2 that persists after PRC2 has dissociated from H3K27M-containing chromatin [104]. This study did not identify the molecular mechanism, but a subsequent work showed that H3K27M reduces the automethylation of PRC2 [105], which is required for high methyltransferase activity [105,106]. This might provide one explanation for a lasting effect on PRC2 activity. While the molecular mechanism for H3K27M-mediated inhibition of PRC2 is not under consensus at this time, most of the current literature points to the H3K27M onco-histone as an antagonist of gene repression.

## Conclusions

PRC2 *reads* multiple chromatin signals at a given genomic site and a given point in time. PRC2 then expresses this information by *writing* its H3K27 mono-, di- and tri-methyl mark in a context-dependent manner. Therefore, the H3K27 methylation at a specific genomic region integrates information about nucleosome density, levels of activating chromatin modifications (H3K4me3 and H3K36me2/3), levels of repressive marks (H3K27me3, H2AK119ub and 5mC) and transcriptional activity. At a certain point in time, the chromatin state — including all its activating and repressive factors — will set the probability for specific molecular events to take place. Specifically, the presence of H3K27me3 [8–10], exposed nucleosome linker DNA [38,41,58], presence of H2AK119ub [19–24] and high PRC2 concentrations will all increase the probability of H3K27me3 accumulation. Conversely, high RNA expression levels [41,68,79–83], high local concentrations of MLL [45] and presence of H3K4me3 [24,25], high H3K36me3 [25,26] and H3.3-containing nucleosomes [73] will lower the probability for H3K27me3 deposition. Together, the activating and repressive factors set the probability for H3K27me3 deposition and, consequently, for a given gene to be transcribed. Positive and negative feedback loops give the active and repressed states a certain stability over time, with PRC2 being an important cog in this complex machine.

## Perspectives

- PRC2 is central to embryonic development and is dysregulated in cancer and congenital disorders. Emerging evidence indicates that PRC2 has the ability to read molecular cues in its surroundings, which in turn regulate its activity.
- PRC2 is regulated by histone modifications, RNA, nucleosome positioning, chromatin structure, and possibly also DNA methylation and DNA sequence-coded features such as CpG density. These different types of molecular cues allow for context-specific regulation of PRC2.
- Future development of strategies to alter the reading functions of PRC2 might open paths for epigenetic drugs to target PRC2 in a context-specific manner.

## Open Access

Open access for this article was enabled by the participation of Monash University in an all-inclusive *Read & Publish* pilot with Portland Press and the Biochemical Society under a transformative agreement with CAUL.

## Abbreviations

5mC: 5-methyl-cytosine
CpG: 5′-C-phosphate-G-3′
DNMT: DNA methyltransferase
EED: Embryonic ectoderm development protein
EZH2: Enhancer of zeste homolog 2
JARID2: Jumonji/ARID domain-containing protein 2
MLL: mixed lineage leukemia
MTF2: Metal regulatory transcription factor 2
PcG: polycomb-group
PCL: Polycomb-like
PHFs: PHD finger proteins
PRCs: Polycomb repressive complexes
SAM: S-adenosyl-L-methionine
SET: Su(var)3–9, Enhancer of Zeste and Trithorax
